# Effects of oleanolic acid administration on renal NF-kB, IL-18, IL-6, YKL-40, and KIM-1 in experimental diabetic rats

**DOI:** 10.22038/IJBMS.2023.71321.15504

**Published:** 2023

**Authors:** Hatice Iskender, Eda Dokumacioglu, Armagan Hayirli, Kubra Asena Terim Kapakin, Ismail Bolat, Esra Manavoglu Kirman

**Affiliations:** 1 Artvin Coruh University, Faculty of Health Sciences, Department of Nutrition and Dietetics, Artvin 08000, Turkey; 2 Department of Animal Nutrition and Nutritional Disorders, Faculty of Veterinary Medicine, Ataturk University, Erzurum 25240, Turkey; 3 Department of Pathology, Faculty of Veterinary Medicine, Ataturk University, Erzurum 25240, Turkey

**Keywords:** Cytokines, Diabetes, KIM-1, Oleanolic acid, YKL-40

## Abstract

**Objective(s):**

Neuropathy, retinopathy, and nephropathy, known as the triopathy of diabetes, are the consequences of microvascular complications of diabetes. The present study aimed to investigate the potential protective effects of oleanolic acid (OA) administration against diabetic nephropathy considering biochemical and histopathological parameters.

**Materials and Methods:**

The rats with fasting blood glucose levels of 200 mg/dl and above were considered diabetic after induction of diabetes via injecting STZ. The other half of the rats were not injected with STZ (healthy rats). Both healthy and diabetic rats were then divided randomly into two subgroups to be administered with either OA (5 mg/kg) with 1 ml tap water by oral gavage or 1 ml tap water in the same route for 21 days. Serum urea-N, Ca, P, and Mg as well as renal tissue MDA, SOD, NF-κB, IL-6, IL-18, AMPK, YKL-40, and KIM-1 levels were measured.

**Results:**

OA administration partially decreased levels of serum urea-N and P, as well as levels of renal tissue MDA and inflammation markers (NF-κB, IL-6, IL-18, YKL-40, and KIM-1) in the diabetic rats. It also partially increased serum Ca and renal tissue AMPK levels in diabetic rats. These positive effects were also seen in renal tissue histopathology.

**Conclusion:**

OA treatment partially alleviated renal damage inflammatory and oxidative profiles in diabetic rats.

## Introduction

Diabetes Mellitus (DM), is a chronic metabolic disease characterized by hyperglycemia, which occurs as a result of insufficient secretion of insulin from the pancreatic gland and/or inefficacy of insulin hormone at extra-hepatic tissues, causing disorders in carbohydrate, protein, and fat metabolism (1-4). Neuropathy, retinopathy, and nephropathy, known as the triopathy of diabetes, are microvascular complications of diabetes. Diabetic kidney disease is one of the most common microvascular complications of diabetes mellitus and is the leading cause of end-stage renal diseases (5, 6). Many factors contribute to the development of diabetic renal diseases, such as hyperglycemia, hypertension, obesity, sedentary lifestyle, heredity, smoking, and advanced age (7).

Oxidative stress and inflammation are intimately linked with the development of diabetic nephropathy. Increases in oxidative stress can induce the production of inflammatory cytokines that can stimulate the production of free radicals (8-11). Nuclear factor kappa B (NF-κB), a transcription factor regulating cell proliferation and apoptosis, generates a response to oxidative stress and plays a key role in inflammation control (12). More specifically, NF-κB activates mesangial cells, which leads to renal injury. It is known that NF-κB expression is increased in the kidneys of diabetic experimental animals (13). Interleukin-18 (IL-18) is a potent inflammatory cytokine secreted from activated monocytes/macrophages (14) and stimulates the production of other inflammatory cytokines including IL-1, tumor necrosis factor-alpha (TNF-α), and IL-6 (15, 16). IL-18 is constitutively expressed in renal tubular epithelia. 5′-adenosine monophosphate (AMP)-activated protein kinase (AMPK) plays an important role in maintaining cell energy balance and regulates many different metabolic and physiological processes. The metabolic and physiological functions of AMPK are very important in preventing and healing many diseases such as obesity, inflammation, diabetes, and cancer (17). Chitinase-like protein (YKL-40), also known as human cartilage glycoprotein-39 (HCgp-39), is a 40 KDa heparin- and chitin-binding glycoprotein and is secreted by chondrocytes, synovial cells, and neutrophils (18). As an inflammatory marker, YKL-40 is associated with DM (19). Kidney Injury Molecule-1 (KIM-1) is expressed on renal proximal tubule epithelial cells and has a significant role in renal regeneration processes (20).

At the onset and the course of nephropathy, one of the microvascular complications of diabetes can be substantially treated by various interventions (21). In recent years, there has been a growing interest in the therapeutic effects of compounds that are naturally found in plants and secreted as secondary metabolites from plants for diabetes, obesity, and cancer as well as daily use for well-being. OA is an active pentacyclic triterpenoid compound isolated from more than 120 plant species, including many nutritious plants and medicinal aromatic plants (22-24). Various studies revealed significant biological benefits of OA due to its antioxidant, antidiabetic, anti-inflammatory, antiparasitic, and antimicrobial activities (25, 26). The present study aimed to investigate the potential protective effects of OA treatment in an experimental diabetic nephropathy model.

## Materials and Methods

***Experimental animals***

The rats were obtained from Medical Experimental Application and Research Centre upon approval by Ataturk University Experimental Animals Local Ethics Committee (No. 236, dated 2021). Twenty-eight male 6-8 week old wistar rats were housed in transparent polyethylene cages and fed *ad libitum* water and a pelleted chow diet. The room was furnished with artificially controlled temperature (23±2 ^°^C), illumination (12:12 hr photoperiod), and humidity (55±5%). After a 1-wk adaptation period, a single dose of 50 mg/kg of streptozotocin (STZ) (Sigma Chemical Co., St. Louis, MO, USA) solution prepared in citrate buffer was administered intraperitoneally to the abdominal cavity of half of the rats for diabetes induction. An average of 72 hrs later, fasting blood glucose was measured by a glucometer from the blood sample taken from the tail vein (27). The rats with fasting blood glucose levels ≥ 200 mg/dl were considered diabetic. The other half of the rats were not injected with STZ (healthy rats). Both healthy and diabetic rats were then divided randomly into two subgroups to be administered either OA (5 mg/kg, Sigma Chemical Co.) in 1 ml tap water by oral gavage or 1 ml tap water in the same route for 21 days (28).

At the end of the experiment, intracardiac blood samples were taken from the rats. Before blood sampling rats were administered ketamine (80 mg/kg; Ketalar®, 50 mg/ml, Eczacıbaşı, Istanbul, Turkey) and xylazine (10 mg/kg; Rompun®, 2%, Bayer, Istanbul, Turkey), and then they were sacrificed. Blood and kidney samples were collected for biochemical analyses and histopathologic evaluations.

***Biochemical analysis***

Blood samples were centrifuged at 4,000 *xg* for 15 min and the sera were stored at -80 ^°^C for measurements of blood urea nitrogen (BUN), phosphor (P), calcium (Ca), and magnesium (Mg) in an autoanalyzer (RX Monaco; Randox Laboratories Ltd., County Antrim, UK).

Kidney tissue malondialdehyde (MDA) levels were measured to assess lipid peroxidation level (29), based on reaction with thiobarbituric acid at 90-95 ^°^C to yield a pink-colored chromogen. After 15 min, the absorbance values of the rapidly cooled samples were read spectrophotometrically at 532 nm. The MDA level was expressed as nmol/g tissue protein. Kidney tissue superoxide dismutase (SOD) level was determined based on the reduction of nitroblue tetrazolium by the xanthine-xanthine oxidase system (30). The SOD activity was expressed as U/g tissue protein.

Kidney tissue NF-κB (Elabscience Biotechnology Inc., Houston, TX, USA), IL-6 (Elabscience Biotechnology Inc.), IL-18 (ELK Biotechnology CO., Ltd., Wuhan East Lake Hi-Tech Development Zone, Hubei, P.R.C), AMPK (Biocompare, South San Francisco, CA, USA), NF-κB (Elabscience Biotechnology Inc.), YKL-40 (ELK Biotechnology CO., Ltd.), and KIM-1 (ELK Biotechnology CO., Ltd.) levels were measured by the ELISA method, based on a specific antigen and antibody reaction. An enzyme is used as a marker in the preparation of the labeled conjugate. After completion of the reaction, separation is achieved by adding a substrate to the medium, and enzyme activity is measured spectrophotometrically. The KIM-1, IL-18, IL-6, and NF-κB results were expressed as pg/ml, whereas the YKL-40 and AMPK results were expressed as ng/ml.

***Histopathological examination and image analysis***

The kidney tissue samples were fixed in 10% buffered formalin and routinely processed for histological examination by embedding them in paraffin wax. Tissue sections were cut in 4 μm thickness and stained by the Haematoxylin Eosin (H&E) (31). Tissue sections were then evaluated by high-power light microscopic examination using an Olympus Bx51 with a DP72 camera system (Olympus Corp., Tokyo, Japan). Each specimen was examined in 10 randomly selected areas of approximately an X40 objective. The scores were derived semi-quantitatively: Grade 0=-(negative); Grade 1=+1 (mild); Grade 2=+2 (moderate); Grade 3=+3 (severe); Grade 4=+4 (most severe) (32).

***Statistical analysis***

Data were analyzed on commercial software (MedCalc, Version 13.2.2; MedCalc, Ostend, Belgium). The normality of data was evaluated using a Kolmogorov-Smirnov test. Continuous data (biochemistry) were analyzed by 2-way ANOVA to test the main effects of health status (HS; healthy *vs* diabetic) and OA treatment (TRT; not OA administered *vs* OA administered) as well as their interaction using the General Linear Model Procedure [Y_ijk_=HS_i_ x TRT_j_+(HS x TRT)_ij_+e_ijk_]. The group mean differences were attained by the LSD option. Discrete data (histopathology) were subjected to the Mann-Whitney U test. Statistical significance is declared* P≤0.05*.

## Results

***Biochemistry***

Diabetes significantly increased serum BUN (189%) and P (45%) concentrations and decreased serum Ca concentration by 20% in rats compared to the healthy group, but did not affect serum Mg concentration (Table 1). Significant health status by treatment interaction showed that OA administration decreased serum BUN and P concentrations and increased serum Ca concentration in the diabetic rats, while not affecting the healthy rats.

Diabetes induction resulted in a 104% increase in renal tissue MDA level and a 20% decrease in renal tissue SOD level. OA administration did not affect renal tissue MDA level in the healthy rats but tended to decrease it in the diabetic rats. There was a similar effect of OA administration on renal tissue SOD levels in healthy and diabetic rats (Table 2).

Except for a decrease in renal tissue AMPK levels, there were more than double increases in renal tissue NF-κB, IL-6, IL-18, YKL-40, and KIM-1 levels in the diabetic rats compared to the healthy rats. OA administration decreased renal tissue NF-κB, IL-6, IL-18, YKL-40, and KIM-1 levels and increased renal tissue AMPK levels in diabetic rats. However, OA administration was completely effective to alleviate these increases and decreases to reach their levels for the healthy rats (Table 3).

***Histopathology***

**Figure 1:**
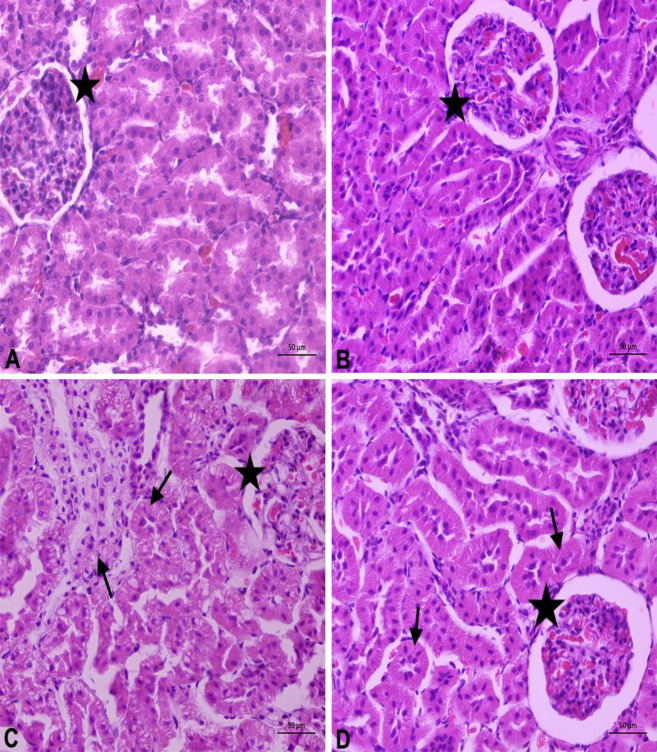
Effect of oleanolic acid administration on histopathology of kidney tissue in diabetic rats

Significant scores for degeneration, necrosis, and presence of inflammatory cells in kidney tissues indicated the success of DM induction (Table 4). There were no histopathological findings in the healthy rats (Figure 1. A-B). In kidney tissues of the diabetic rats, atrophy in glomeruli and tubules as well as degenerative changes, especially vacuolar degenerations and partial necrosis in tubules were observed (Figure 1C). There were also dilatations in the Bowman’s capsule of the glomeruli and the lumens of some tubules, and lymphocyte cell infiltrations in the interstitial area, especially in the periglomerular region. OA administration was partially effective to restore kidney tissue histopathology of the diabetic rats (Table 4; Figure 1D).

**Table 1:** Effect of oleanolic acid administration on serum metabolites in diabetic rats

Groups^1^		Parameters^2^			
Status	Treatment	BUN (mg/dl)	P (mg/dl)	Ca (mg/dl)	Mg (mg/dl)
HEA		14.9±0.7	3.74±0.14	8.79±0.18	2.06±0.07
DM		43.0±6.2	5.43±0.52	7.41±0.46	1.91±0.07
	No	37.7±7.4	5.55±0.47	7.51±0.47	2.02±0.08
	Yes	20.2±1.9	3.62±0.18	8.69±0.22	1.94±0.06
HEA	No	14.7±1.1^b^	3.96±0.15^b^	8.98±0.28^a^	2.13±0.11
HEA	Yes	15.1±1.0^b^	3.53±0.22^b^	8.59±0.23^a^	1.99±0.09
DM	No	60.7±7.6^a^	7.15±0.31^a^	6.04±0.38^b^	1.91±0.10
DM	Yes	25.3±2.3^b^	3.71±0.29^b^	8.79±0.39^a^	1.90±0.09
ANOVA		------------------------------ p< ------------------------------			
Status		0.0001	0.0001	0.0004	0.1410
Treatment		0.0002	0.0001	0.0016	0.4331
Status*Treatment		0.0002	0.0001	0.0001	0.5204

^1 ^HEA=Healthy rats; DM=Diabetic rats administered a single dose of 50 mg/kg of streptozotocin. No=Rats given 1 ml tap water by oral gavage; Yes=Rats treated with OA in 1 ml tap water by oral gavage

^2 ^BUN=Blood urea nitrogen; Ca=Calcium; Mg=Magnesium; P=Phosphorus. Data are the least square means±standard error of mean from 2-way ANOVA. Different superscripts within columns differ in the interaction rows (*P≤0.05*)

**Table 2:** Effect of oleanolic acid administration on kidney tissue malondialdehyde and superoxide dismutase levels in diabetic rats

Groups^1^		Parameters^2^	
Status	Treatment	MDA (nmol/g)	SOD (U/gr)
HEA		12.3±1.2	81.9±11.6
DM		25.0±1.9	65.5±9.0
	No	21.8±2.7	47.7±4.5
	Yes	15.5±1.5	99.8±10.0
HEA	No	12.9±2.1^c^	55.5±7.2^b^
HEA	Yes	11.7±1.3^c^	108.5±17.2^a^
DM	No	30.8±1.2^a^	40.0±4.0^b^
DM	Yes	19.2±1.8^b^	91.0±10.8^a^
ANOVA		----------------------- p< -----------------------	
Status		0.0001	0.1473
Treatment		0.0007	0.0001
Status*Treatment		0.0042	0.9248

^1 ^HEA=Healthy rats; DM=Diabetic rats administered a single dose of 50 mg/kg of streptozotocin. No=Rats given 1 ml tap water by oral gavage; Yes=Rats treated with OA in 1 ml tap water by oral gavage

^2 ^MDA=Malondialdehyde; SOD=Superoxide dismutase. Data are the least square means±standard error of mean from 2-way ANOVA. Different superscripts within columns differ in the interaction rows (*P≤0.05*)

**Table 3:** Effect of oleanolic acid administration on kidney tissue NF-κB/IL-6/IL-18 and YKL-40/KIM-1 levels in the diabetic rats

Groups^1^		Parameters^2^					
Status	Treatment	NF-κB(pg/ml)	IL-6(pg/ml)	IL-18 (pg/ml)	AMPK (ng/ml)	YKL-40 (ng/ml)	KIM-1 (pg/ml)
HEA		3.03±0.23	4.53±0.36	46.5±2.0	47.1±1.5	6.92±0.58	511±32
DM		5.75±0.49	10.55±0.93	131.0±21.0	31.6±1.5	12.60±0.80	1218±142
	No	5.05±0.68	8.66±1.36	120.0±23.0	37.0±2.6	10.50±1.30	1085±169
	Yes	3.73±0.22	6.42±0.58	57.3±7.2	41.6±2.4	8.96±0.66	644±65
HEA	No	2.73±0.31^c^	4.33±0.44^c^	45.2±3.4^b^	45.9±1.0^a^	6.51±1.09^c^	541±62^bc^
HEA	Yes	3.34±0.33^bc^	4.72±0.60^c^	47.8±2.1^b^	48.1±2.9^a^	7.33±0.48^c^	481±17^c^
DM	No	7.37±0.37^a^	13.00±1.26^a^	195.0±19.0^a^	28.2±1.4^c^	14.60±1.00^a^	1629±147^a^
DM	Yes	4.12±0.21^b^	8.11±0.40^b^	66.7±13.7^b^	35.1±1.8^b^	10.60±0.90^b^	807±95^b^
ANOVA		-------------------------------------------------------------- p< ---------------------------------------------------------------					
Status		0.0001	0.0001	0.0001	0.0001	0.0001	0.0001
Treatment		0.0003	0.0067	0.0001	0.0248	0.0948	0.0001
Status*Treatment		0.0001	0.0019	0.0001	0.2286	0.0141	0.0004

**Table 4:** Effect of oleanolic acid administration on histopathology of kidney tissue in diabetic rats

Groups^1^		Parameters^2^		
Status	Treatment	Degeneration	Necrosis	Inflammatory cells
HEA		0 (0-1)	0 (0-0)	0 (0-0)
DM		2 (1-3)	1 (0-3)	1 (0-2)
	No	1,5 (0-3)	0,5 (0-3)	0 (0-2)
	Yes	1 (0-3)	0 (0-2)	0 (0-1)
HEA	No	0 (0-1)^c^	0 (0-0)^b^	0 (0-0)^c^
HEA	Yes	1 (0-1)^c^	0 (0-0)^b^	0 (0-0)^c^
DM	No	2 (2-3)^a^	1 (1-3)^a^	2 (0-2)^a^
DM	Yes	1 (1-3)^b^	0 (0-2)^b^	1 (0-1)^b^
ANOVA		--------------------------------- p< ---------------------------------		
Status		0.0001	0.0001	0.0001
Treatment		0.2180	0.0120	0.0254
Status*Treatment		0.0184	0.0120	0.0254

^1 ^HEA=Healthy rats; DM=Diabetic rats administered with a single dose of 50 mg/kg of streptozotocin. No=Rats given 1 ml tap water by oral gavage; Yes=Rats treated with OA in 1 ml tap water by oral gavage

^2^ Data are the median (minimum-maximum) from the Mann-Whitney U test. Grade 0=Negative; Grade 1=Mild; Grade 2=Moderate; Grade 3=Severe; Grade 4=Most severe (Apaydin-Yildirim *et al*.). Different superscripts within columns differ in the interaction rows (*P≤0.05*)

H&E, Bar: 50 μm

A-B) No histopathological lesions in the healthy rats not administered with oleanolic acid (OA) and administered with OA

C) Degenerative changes, especially vacuolar degenerations and partial necrosis in some tubules in diabetic rats not administered with OA

D) Moderate degenerative changes in kidney tissue in the diabetic rats administered with OA

## Discussion

Various treatment opportunities are available for the treatment and follow-up of diabetes, including pharmacological approaches and non-pharmacological approaches such as dieting, exercising, and losing weight. Diabetic patients generally prefer complementary and alternative medicine, as they consider the minimal or negligible side effects of these treatments (33). In recent years, there is a growing interest in using natural components of herbal origin, considering the increasing number of diabetic cases, potential adverse effects of the relevant medicines, patients’ prejudices about long-term use of medicines, and increasing drug resistance. The pharmacological effects of natural metabolites such as OA attracted great interest in recent years, and they were pointed out as effective therapeutic agents used in phytotherapy and treatment. This study aimed to evaluate the effects of OA administration on kidney tissue parameters of rats with STZ-induced DM.

Diabetic nephropathy begins with increased glomerular permeability against proteins, then progresses to microalbuminuria and azotemia, and eventually results in renal failure (34). Serum urea-N (or BUN) levels are the most common indicators used in the evaluation of renal function. Disruption of Ca, P, and Mg homeostasis is common in DM and may be associated with increased morbidity and mortality because these elements play a structural role and participate in the glucose transport system (35, 36). Serum urea-N and mineral profile, except for Mg, is compatible with literature dealing with diabetic conditions, which was completely corrected by OA administration (Table 1).

Diabetes is a serious cause of oxidative stress that is responsible for diabetic complications. ROS damage the cell membrane, inactivate antioxidant enzymes, alter endogenous antioxidant gene expression, and contribute to the pathogenesis of DM (37). In some studies, on oxidative damage in diabetes, MDA levels increased due to increased ROS in various tissues of diabetic rats but decreased significantly in diabetic groups administered plant extracts or antioxidant molecules (38, 39). In this experiment, OA administration partially reduced renal tissue MDA levels, which was increased due to DM induction (Table 2).

Inflammatory processes are closely associated with pathological mechanisms such as glycotoxicity, lipotoxicity, and oxidative stress. Stimuli including bad eating habits, increased abdominal obesity, sedentary lifestyle, smoking, and alcohol consumption increase the release of proinflammatory factors such as IL-6 and IL-18 from various tissues. This can cause chronic low-grade inflammation, identified as a risk factor for insulin resistance, cellular dysfunction, and ultimately the development of diabetes (40, 41). Oxidative stress can increase cytokine production via several different mechanisms. Oxygen derivatives, acting as second messengers, activate the transcription factors NF-κB and lead to the transcription of genes encoding cytokines (42). NF-κB regulates multiple target genes involved in cell growth and survival. Inhibition of NF-κB, which induces inflammation, alleviates diabetes and reduces hyperglycemia and insulin resistance. In the development of DM, oxidative stress-mediated NF-κB activation is very likely the central signal triggering and propagating the autoimmune process, resulting in pancreatic β-cell death (43). Studies revealed the important role of NF-κB in the development of diabetic nephropathy (44, 45). In DM, oxidative stress causes tissue damage by activating NF-κB. NF-κB inhibitors, alleviating the effect of oxidative stress and preventing this tissue damage, can be used in the treatment of DM (46).

Inflammation plays a key role in the pathogenesis of diabetic nephropathy. Cytokines are produced by a wide variety of cells in the body, playing an important role in many physiological responses that have therapeutic potential. Renal IL-6 levels are positively related to mesangial proliferation and tubular atrophy in diverse models of renal disease, supporting the role of IL-6 in the progression of renal disease (41, 47). It was reported in the literature that the increase in IL-18 levels was parallel with the increase in IL-6 levels, in diabetic nephropathy (15, 48). In our study, the levels of NF-κB, IL-6, and IL-18 increased in diabetic rats, which were partially reversed by OA administration (Table 3).

Numerous different agents and methods are under investigation and development for reducing or eliminating inflammation and oxidative stress in diabetes, stopping the progression of diabetes, preventing its macro and micro complications, or lowering blood glucose levels. In order to achieve these effects via natural products, studies examining the activities of plant-derived compounds and extracted purified substances are becoming more popular today. OA was the target compound in the study, considering that it may be a natural anti-diabetic substance. Various inflammatory cytokines, including IL-6 and TNF-α, have been shown to activate NF-κB to cause insulin resistance. OA has anti-inflammatory effects. It not only reduced the content of inflammatory cytokines but also reduced the expression of NF-κB (49). In our study, OA administration decreased NF-κB, IL-6, and IL-18 levels (Table 3). AMPK is highly sensitive to the cellular level of ROS. Also, oxidative stress can cause intracellular ATP depletion. At the same time, recent studies revealed that ROS stimulated AMPK activation even without a reduction in cellular ATP. It was observed that AMPK was activated under oxidative stress conditions by oxidative modification of the AMPK α-subunit. As can be seen, ROS can induce AMPK activation, without decreasing ATP levels in the cell (50, 51). OA treatment increased AMPK values, which decreased in diabetic rats as compared to healthy rats. The AMPK is a cellular energy sensor with many different activators and is involved in metabolic functions in the body such as autophagy, insulin-independent GLUT4 translocation, and fatty acid oxidation. In the literature, it was reported that AMPK can be a potential alternative to current treatment options and AMPK has an important role in the treatment of diseases such as prediabetes, diabetes, and obesity, in case it is activated by various natural activators having antioxidant properties (52, 53). It seems OA behaves as an AMPK activator since it is secreted by many cells involved in infectious processes and participated in chronic inflammation, YKL-40 is recognized as an acute-phase protein and reported as an early marker of chronic low-grade inflammation (19, 54). In the literature (Table 3), serum YKL-40 levels seem to increase in parallel with the development of DM and seem to be elevated in DM patients when compared with the control groups (55). KIM-1 plays a significant role in renal regeneration processes. This is because KIM-1, being a differentiation and proliferation marker, is totally undetected in the normally functioning renal system and only expressed in tubular cells after renal injury (15). Kapoula *et al*. (56) provide additional data about the role of two biomarkers, KIM-1 and YKL-40, in the development of early diabetic nephropathy, with both being promising biomarkers in the diagnosis of the disease as they can be detectable in early stages and subclinical diseases. In our study, KIM-1 and YKL-40 levels increased in diabetic rats, while they decreased by OA administration. Diabetic nephropathy is characterized by morphological and ultrastructural changes in the kidney including expansion of the molecular matrix and loss of the charge barrier on the glomerular basement membrane (57, 58). In our study, OA administration ameliorated these lesions in diabetic rats (Figure 1. D). There is a close association between oxidative stress and inflammation in diabetes and we hypothesize that an increase in oxidative stress-derived inflammation is a major mechanism in the pathogenesis and progression of diabetic nephropathy.

## Conclusion

Kidney damage increased in rats with STZ-induced DM. However, the kidney damage partially improved in diabetic rats administered with OA. It can be considered that OA treatment may be beneficial in preventing kidney damage in conditions such as diabetes and diabetic nephropathy. These findings need to be supported by further experimental studies considering OA supplementation at different doses and times as well as clinical studies.

## Authors’ Contributions

H I and E D designed the study and took part in data interpretation and article writing; E D and H I contributed to article writing; I B and E M K contributed to animal experiments; H I and E D contributed to biochemical analysis and interpretation. K T K and I B contributed to histopathological analysis, and A H contributed to statistical analysis.

## Conflicts of Interest

No potential conflict of interest was reported by the authors. 
